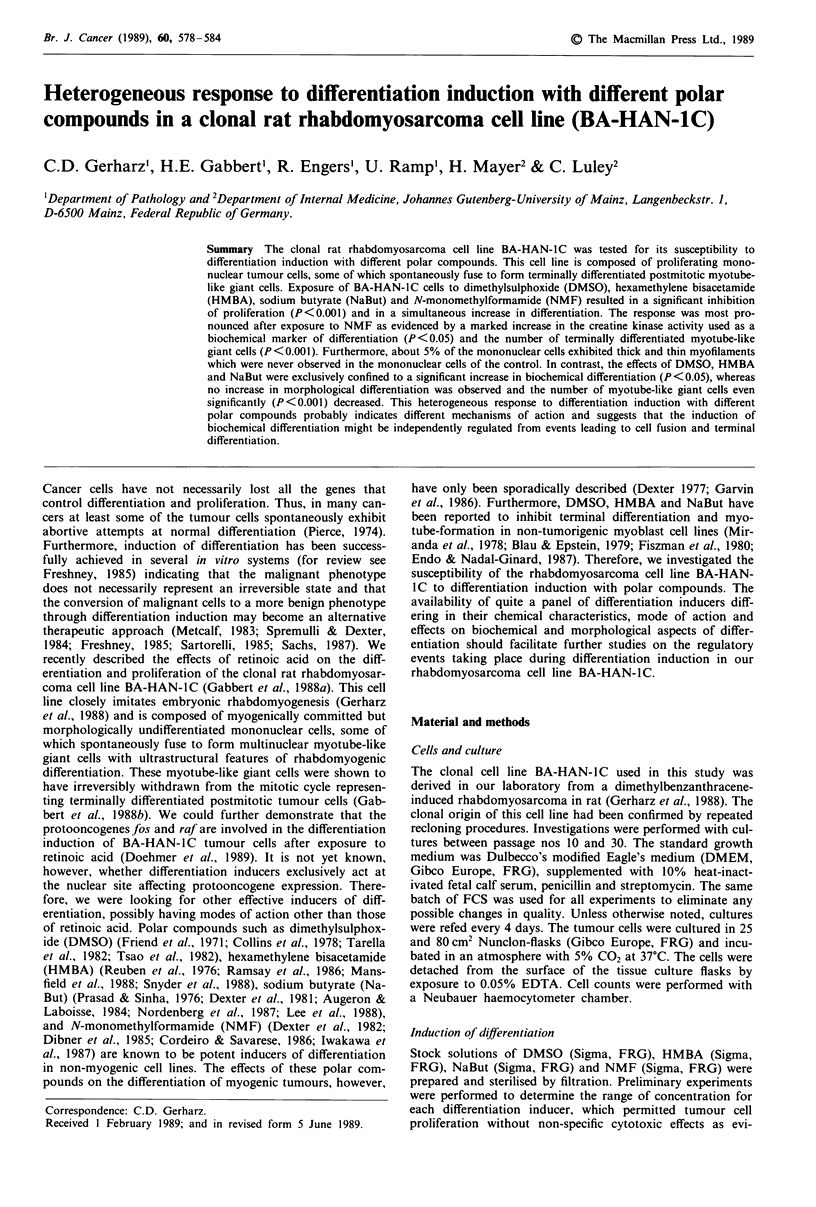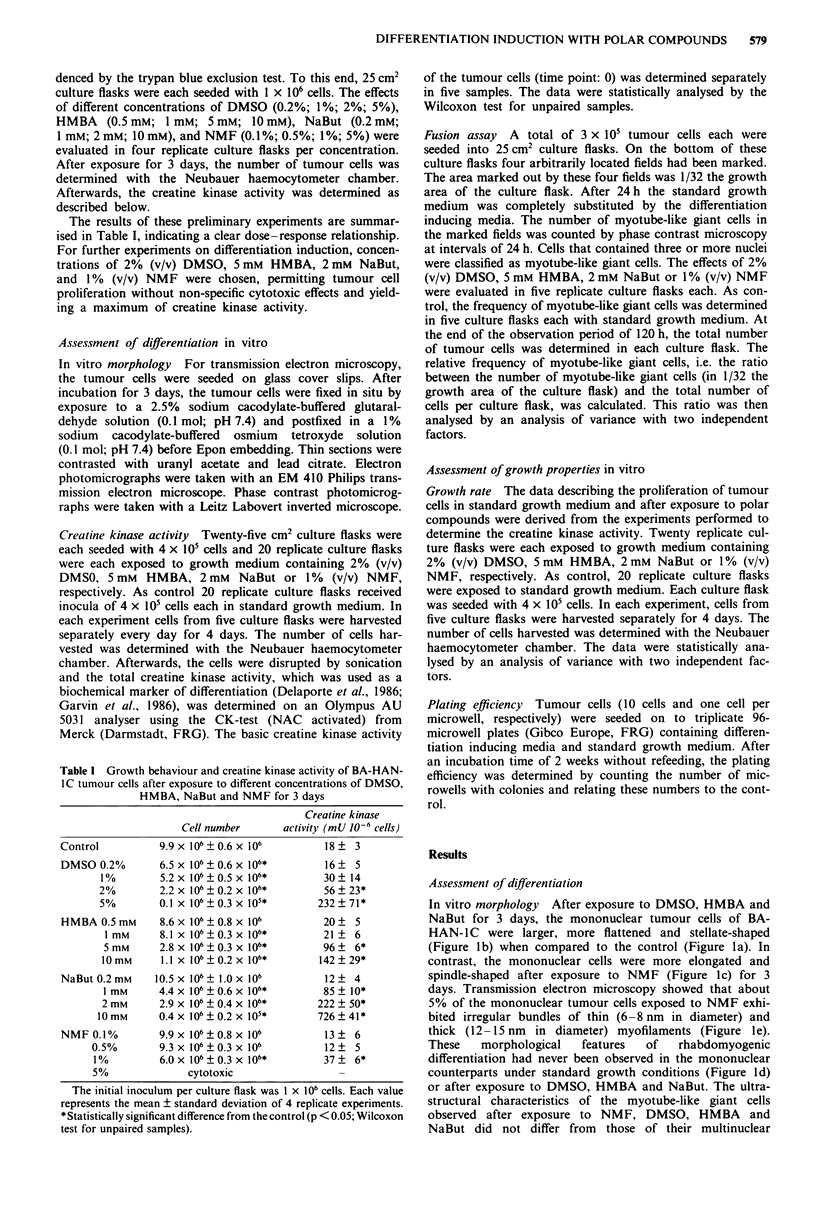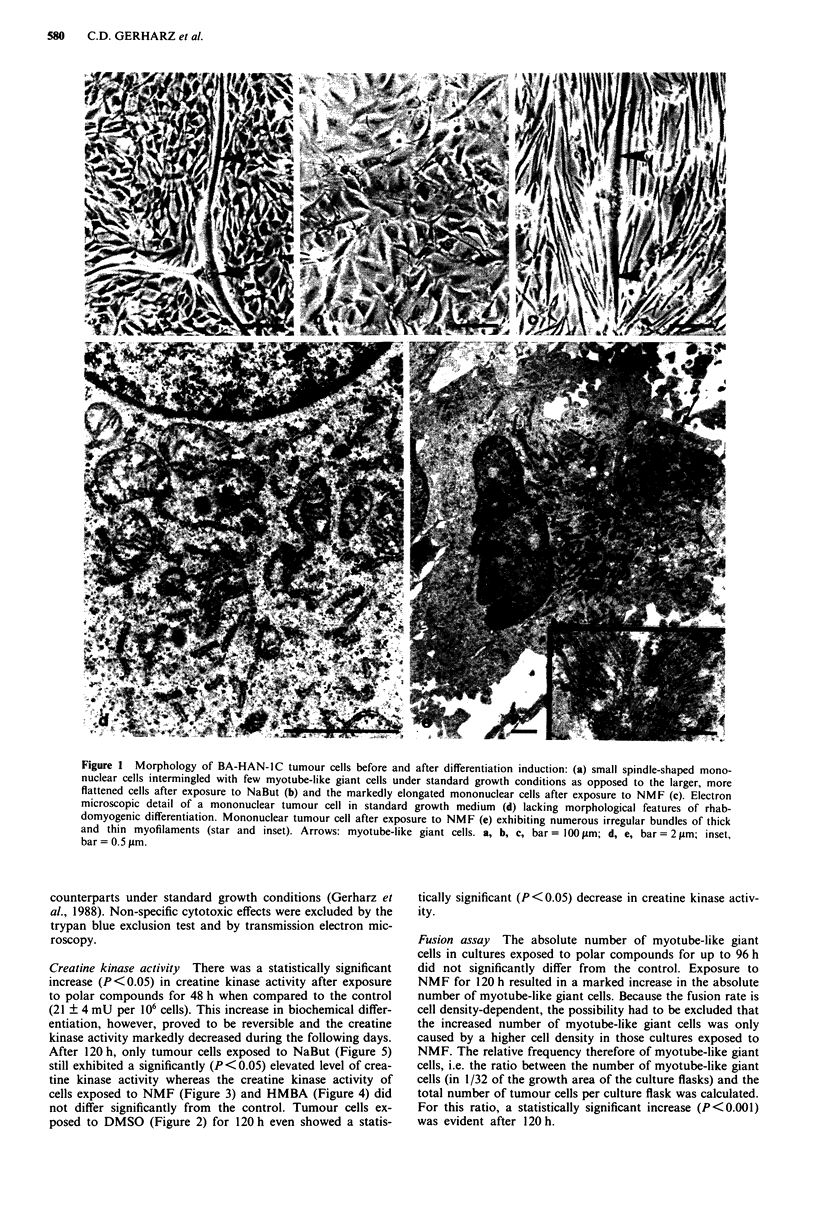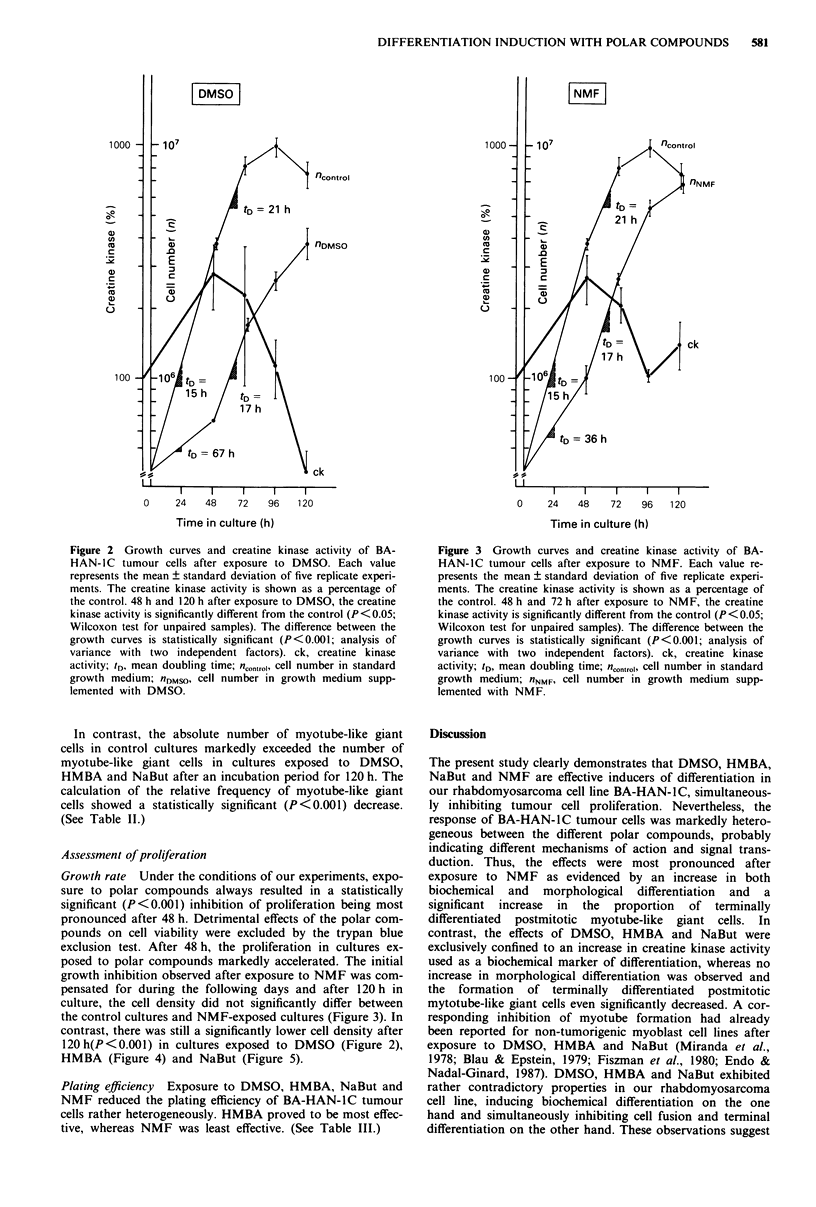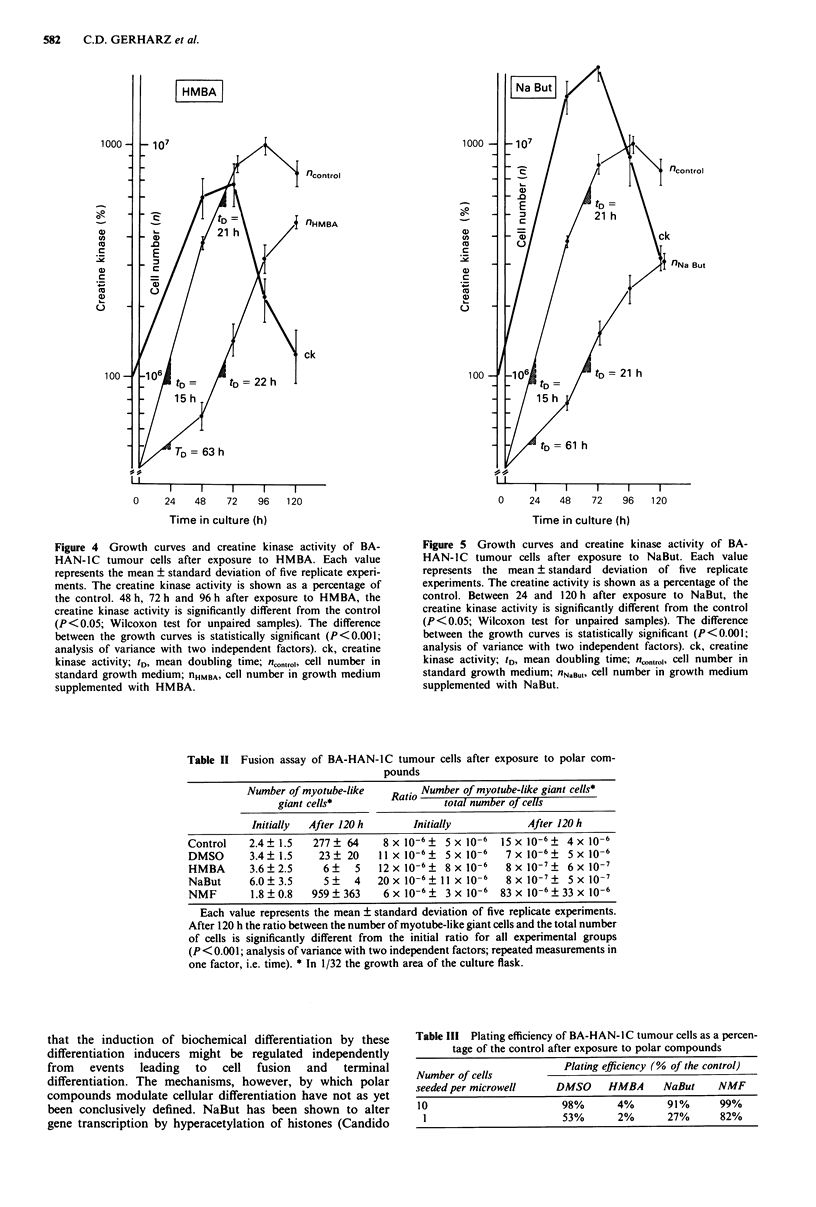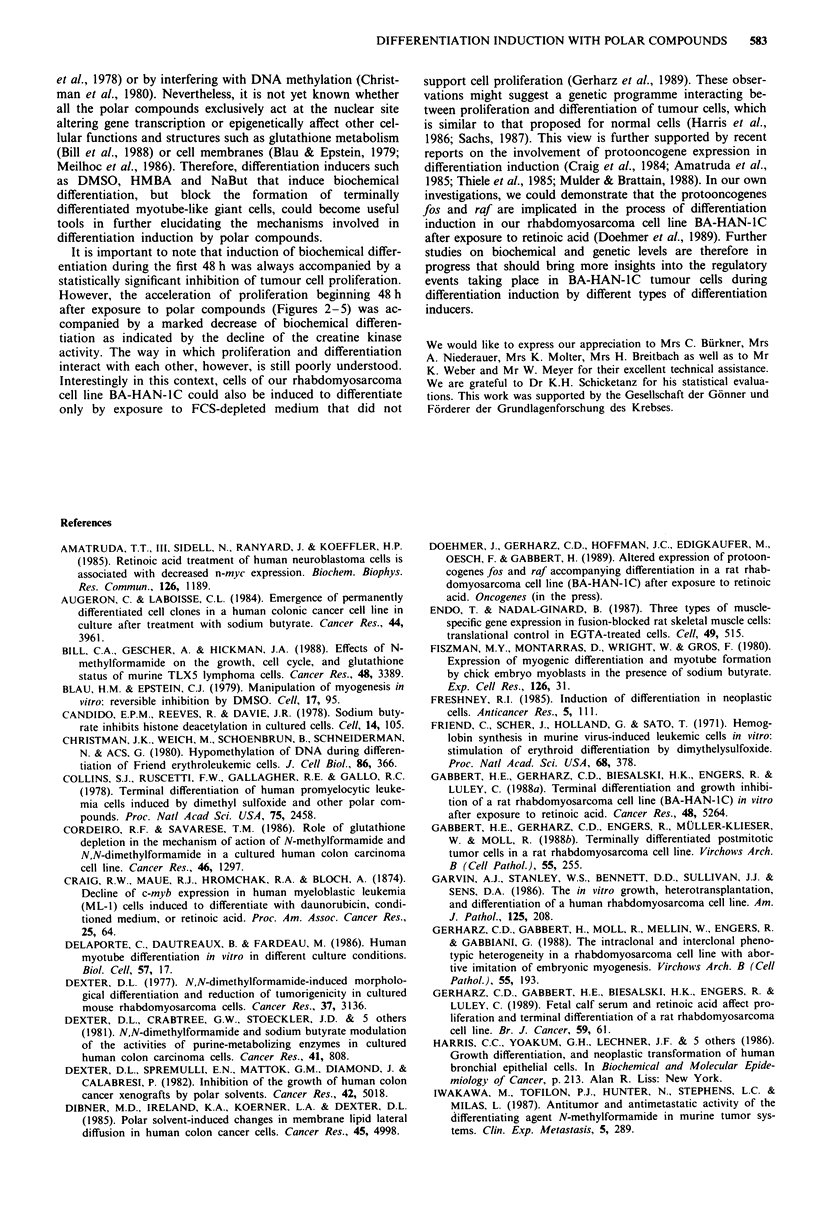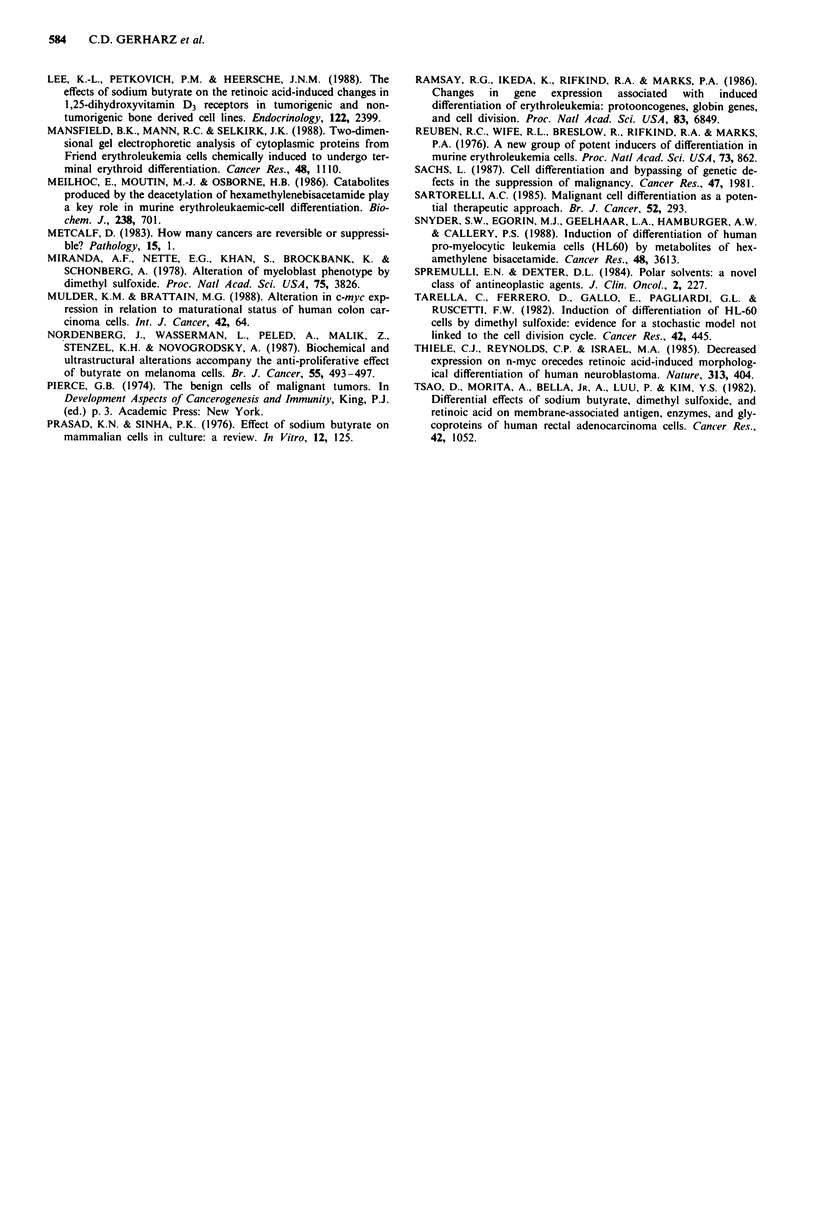# Heterogeneous response to differentiation induction with different polar compounds in a clonal rat rhabdomyosarcoma cell line (BA-HAN-1C).

**DOI:** 10.1038/bjc.1989.317

**Published:** 1989-10

**Authors:** C. D. Gerharz, H. E. Gabbert, R. Engers, U. Ramp, H. Mayer, C. Luley

**Affiliations:** Department of Pathology, Johannes Gutenberg-University of Mainz, Federal Republic of Germany.

## Abstract

**Images:**


Br. J. Cancer (1989), 60, 578-584 © The Macmillan Press Ltd., 1989

### Heterogeneous response to differentiation induction with different polar compounds in a clonal rat rhabdomyosarcoma cell line (BA-HAN-1C)

C.D. Gerharz[1], H.E. Gabbert[1], R. Engers[1], U. Ramp[1], H. Mayer[2] & C. Luley[2]

[1]*Department of Pathology and* [2]*Department of Internal Medicine, Johannes Gutenberg-University of Mainz, Langenbeckstr. 1, D-6500 Mainz, Federal Republic of Germany.*

**Summary** The clonal rat rhabdomyosarcoma cell line BA-HAN-1C was tested for its susceptibility to differentiation induction with different polar compounds. This cell line is composed of proliferating mononuclear tumour cells, some of which spontaneously fuse to form terminally differentiated postmitotic myotube-like giant cells. Exposure of BA-HAN-1C cells to dimethylsulphoxide (DMSO), hexamethylene bisacetamide (HMBA), sodium butyrate (NaBut) and *N*-monomethylformamide (NMF) resulted in a significant inhibition of proliferation ($P<0.001$) and in a simultaneous increase in differentiation. The response was most pronounced after exposure to NMF as evidenced by a marked increase in the creatine kinase activity used as a biochemical marker of differentiation ($P<0.05$) and the number of terminally differentiated myotube-like giant cells ($P<0.001$). Furthermore, about 5% of the mononuclear cells exhibited thick and thin myofilaments which were never observed in the mononuclear cells of the control. In contrast, the effects of DMSO, HMBA and NaBut were exclusively confined to a significant increase in biochemical differentiation ($P<0.05$), whereas no increase in morphological differentiation was observed and the number of myotube-like giant cells even significantly ($P<0.001$) decreased. This heterogeneous response to differentiation induction with different polar compounds probably indicates different mechanisms of action and suggests that the induction of biochemical differentiation might be independently regulated from events leading to cell fusion and terminal differentiation.

Cancer cells have not necessarily lost all the genes that control differentiation and proliferation. Thus, in many cancers at least some of the tumour cells spontaneously exhibit abortive attempts at normal differentiation (Pierce, 1974). Furthermore, induction of differentiation has been successfully achieved in several *in vitro* systems (for review see Freshney, 1985) indicating that the malignant phenotype does not necessarily represent an irreversible state and that the conversion of malignant cells to a more benign phenotype through differentiation induction may become an alternative therapeutic approach (Metcalf, 1983; Spremulli & Dexter, 1984; Freshney, 1985; Sartorelli, 1985; Sachs, 1987). We recently described the effects of retinoic acid on the differentiation and proliferation of the clonal rat rhabdomyosarcoma cell line BA-HAN-1C (Gabbert *et al*., 1988*a*). This cell line closely imitates embryonic rhabdomyogenesis (Gerharz *et al*., 1988) and is composed of myogenically committed but morphologically undifferentiated mononuclear cells, some of which spontaneously fuse to form multinuclear myotube-like giant cells with ultrastructural features of rhabdomyogenic differentiation. These myotube-like giant cells were shown to have irreversibly withdrawn from the mitotic cycle representing terminally differentiated postmitotic tumour cells (Gabbert *et al*., 1988*b*). We could further demonstrate that the protooncogenes *fos* and *raf* are involved in the differentiation induction of BA-HAN-1C tumour cells after exposure to retinoic acid (Doehmer *et al*., 1989). It is not yet known, however, whether differentiation inducers exclusively act at the nuclear site affecting protooncogene expression. Therefore, we were looking for other effective inducers of differentiation, possibly having modes of action other than those of retinoic acid. Polar compounds such as dimethylsulphoxide (DMSO) (Friend *et al*., 1971; Collins *et al*., 1978; Tarella *et al*., 1982; Tsao *et al*., 1982), hexamethylene bisacetamide (HMBA) (Reuben *et al*., 1976; Ramsay *et al*., 1986; Mansfield *et al*., 1988; Snyder *et al*., 1988), sodium butyrate (NaBut) (Prasad & Sinha, 1976; Dexter *et al*., 1981; Augeron & Laboisse, 1984; Nordenberg *et al*., 1987; Lee *et al*., 1988), and *N*-monomethylformamide (NMF) (Dexter *et al*., 1982; Dibner *et al*., 1985; Cordeiro & Savarese, 1986; Iwakawa *et al*., 1987) are known to be potent inducers of differentiation in non-myogenic cell lines. The effects of these polar compounds on the differentiation of myogenic tumours, however, have only been sporadically described (Dexter 1977; Garvin *et al*., 1986). Furthermore, DMSO, HMBA and NaBut have been reported to inhibit terminal differentiation and myotube-formation in non-tumorigenic myoblast cell lines (Miranda *et al*., 1978; Blau & Epstein, 1979; Fiszman *et al*., 1980; Endo & Nadal-Ginard, 1987). Therefore, we investigated the susceptibility of the rhabdomyosarcoma cell line BA-HAN-1C to differentiation induction with polar compounds. The availability of quite a panel of differentiation inducers differing in their chemical characteristics, mode of action and effects on biochemical and morphological aspects of differentiation should facilitate further studies on the regulatory events taking place during differentiation induction in our rhabdomyosarcoma cell line BA-HAN-1C.

Correspondence: C.D. Gerharz.
Received 1 February 1989; and in revised form 5 June 1989.

### Material and methods

### *Cells and culture*

The clonal cell line BA-HAN-1C used in this study was derived in our laboratory from a dimethylbenzanthracene-induced rhabdomyosarcoma in rat (Gerharz *et al*., 1988). The clonal origin of this cell line had been confirmed by repeated recloning procedures. Investigations were performed with cultures between passage nos 10 and 30. The standard growth medium was Dulbecco's modified Eagle's medium (DMEM, Gibco Europe, FRG), supplemented with 10% heat-inactivated fetal calf serum, penicillin and streptomycin. The same batch of FCS was used for all experiments to eliminate any possible changes in quality. Unless otherwise noted, cultures were refed every 4 days. The tumour cells were cultured in 25 and 80 $cm^2$ Nunclon-flasks (Gibco Europe, FRG) and incubated in an atmosphere with 5% $CO_2$ at 37°C. The cells were detached from the surface of the tissue culture flasks by exposure to 0.05% EDTA. Cell counts were performed with a Neubauer haemocytometer chamber.

### *Induction of differentiation*

Stock solutions of DMSO (Sigma, FRG), HMBA (Sigma, FRG), NaBut (Sigma, FRG) and NMF (Sigma, FRG) were prepared and sterilised by filtration. Preliminary experiments were performed to determine the range of concentration for each differentiation inducer, which permitted tumour cell proliferation without non-specific cytotoxic effects as evidenced by the trypan blue exclusion test. To this end, 25 $cm^2$ culture flasks were each seeded with $1 \times 10^6$ cells. The effects of different concentrations of DMSO (0.2%; 1%; 2%; 5%), HMBA (0.5 mM; 1 mM; 5 mM; 10 mM), NaBut (0.2 mM; 1 mM; 2 mM; 10 mM), and NMF (0.1%; 0.5%; 1%; 5%) were evaluated in four replicate culture flasks per concentration. After exposure for 3 days, the number of tumour cells was determined with the Neubauer haemocytometer chamber. Afterwards, the creatine kinase activity was determined as described below.

The results of these preliminary experiments are summarised in Table I, indicating a clear dose–response relationship. For further experiments on differentiation induction, concentrations of 2% (v/v) DMSO, 5 mM HMBA, 2 mM NaBut, and 1% (v/v) NMF were chosen, permitting tumour cell proliferation without non-specific cytotoxic effects and yielding a maximum of creatine kinase activity.

### *Assessment of differentiation* in vitro

*In* vitro *morphology* For transmission electron microscopy, the tumour cells were seeded on glass cover slips. After incubation for 3 days, the tumour cells were fixed in situ by exposure to a 2.5% sodium cacodylate-buffered glutaraldehyde solution (0.1 mol; pH 7.4) and postfixed in a 1% sodium cacodylate-buffered osmium tetroxyde solution (0.1 mol; pH 7.4) before Epon embedding. Thin sections were contrasted with uranyl acetate and lead citrate. Electron photomicrographs were taken with an EM 410 Philips transmission electron microscope. Phase contrast photomicrographs were taken with a Leitz Labovert inverted microscope.

*Creatine kinase activity* Twenty-five $cm^2$ culture flasks were each seeded with $4 \times 10^5$ cells and 20 replicate culture flasks were each exposed to growth medium containing 2% (v/v) DMS0, 5 mM HMBA, 2 mM NaBut or 1% (v/v) NMF, respectively. As control 20 replicate culture flasks received inocula of $4 \times 10^5$ cells each in standard growth medium. In each experiment cells from five culture flasks were harvested separately every day for 4 days. The number of cells harvested was determined with the Neubauer haemocytometer chamber. Afterwards, the cells were disrupted by sonication and the total creatine kinase activity, which was used as a biochemical marker of differentiation (Delaporte *et al.*, 1986; Garvin *et al.*, 1986), was determined on an Olympus AU 5031 analyser using the CK-test (NAC activated) from Merck (Darmstadt, FRG). The basic creatine kinase activity

**Table I** Growth behaviour and creatine kinase activity of BA-HAN-1C tumour cells after exposure to different concentrations of DMSO, HMBA, NaBut and NMF for 3 days

| | *Cell number* | *Creatine kinase activity (mU $10^{-6}$ cells)* |
|---|---|---|
| Control | $9.9 \times 10^6 \pm 0.6 \times 10^6$ | $18 \pm 3$ |
| DMSO 0.2% | $6.5 \times 10^6 \pm 0.6 \times 10^6$* | $16 \pm 5$ |
| 1% | $5.2 \times 10^6 \pm 0.5 \times 10^6$* | $30 \pm 14$ |
| 2% | $2.2 \times 10^6 \pm 0.2 \times 10^6$* | $56 \pm 23$* |
| 5% | $0.1 \times 10^6 \pm 0.3 \times 10^5$* | $232 \pm 71$* |
| HMBA 0.5 mM | $8.6 \times 10^6 \pm 0.8 \times 10^6$ | $20 \pm 5$ |
| 1 mM | $8.1 \times 10^6 \pm 0.3 \times 10^6$* | $21 \pm 6$ |
| 5 mM | $2.8 \times 10^6 \pm 0.3 \times 10^6$* | $96 \pm 6$* |
| 10 mM | $1.1 \times 10^6 \pm 0.2 \times 10^6$* | $142 \pm 29$* |
| NaBut 0.2 mM | $10.5 \times 10^6 \pm 1.0 \times 10^6$ | $12 \pm 4$ |
| 1 mM | $4.4 \times 10^6 \pm 0.6 \times 10^6$* | $85 \pm 10$* |
| 2 mM | $2.9 \times 10^6 \pm 0.4 \times 10^6$* | $222 \pm 50$* |
| 10 mM | $0.4 \times 10^6 \pm 0.2 \times 10^5$* | $726 \pm 41$* |
| NMF 0.1% | $9.9 \times 10^6 \pm 0.8 \times 10^6$ | $13 \pm 6$ |
| 0.5% | $9.3 \times 10^6 \pm 0.3 \times 10^6$ | $12 \pm 5$ |
| 1% | $6.0 \times 10^6 \pm 0.3 \times 10^6$* | $37 \pm 6$* |
| 5% | cytotoxic | – |

The initial inoculum per culture flask was $1 \times 10^6$ cells. Each value represents the mean ± standard deviation of 4 replicate experiments. *Statistically significant difference from the control ($p < 0.05$; Wilcoxon test for unpaired samples).

of the tumour cells (time point: 0) was determined separately in five samples. The data were statistically analysed by the Wilcoxon test for unpaired samples.

*Fusion assay* A total of $3 \times 10^5$ tumour cells each were seeded into 25 $cm^2$ culture flasks. On the bottom of these culture flasks four arbitrarily located fields had been marked. The area marked out by these four fields was 1/32 the growth area of the culture flask. After 24 h the standard growth medium was completely substituted by the differentiation inducing media. The number of myotube-like giant cells in the marked fields was counted by phase contrast microscopy at intervals of 24 h. Cells that contained three or more nuclei were classified as myotube-like giant cells. The effects of 2% (v/v) DMSO, 5 mM HMBA, 2 mM NaBut or 1% (v/v) NMF were evaluated in five replicate culture flasks each. As control, the frequency of myotube-like giant cells was determined in five culture flasks each with standard growth medium. At the end of the observation period of 120 h, the total number of tumour cells was determined in each culture flask. The relative frequency of myotube-like giant cells, i.e. the ratio between the number of myotube-like giant cells (in 1/32 the growth area of the culture flask) and the total number of cells per culture flask, was calculated. This ratio was then analysed by an analysis of variance with two independent factors.

### *Assessment of growth properties* in vitro

*Growth rate* The data describing the proliferation of tumour cells in standard growth medium and after exposure to polar compounds were derived from the experiments performed to determine the creatine kinase activity. Twenty replicate culture flasks were each exposed to growth medium containing 2% (v/v) DMSO, 5 mM HMBA, 2 mM NaBut or 1% (v/v) NMF, respectively. As control, 20 replicate culture flasks were exposed to standard growth medium. Each culture flask was seeded with $4 \times 10^5$ cells. In each experiment, cells from five culture flasks were harvested separately for 4 days. The number of cells harvested was determined with the Neubauer haemocytometer chamber. The data were statistically analysed by an analysis of variance with two independent factors.

*Plating efficiency* Tumour cells (10 cells and one cell per microwell, respectively) were seeded on to triplicate 96-microwell plates (Gibco Europe, FRG) containing differentiation inducing media and standard growth medium. After an incubation time of 2 weeks without refeeding, the plating efficiency was determined by counting the number of microwells with colonies and relating these numbers to the control.

### Results

### *Assessment of differentiation*

*In* vitro *morphology* After exposure to DMSO, HMBA and NaBut for 3 days, the mononuclear tumour cells of BA-HAN-1C were larger, more flattened and stellate-shaped (Figure 1b) when compared to the control (Figure 1a). In contrast, the mononuclear cells were more elongated and spindle-shaped after exposure to NMF (Figure 1c) for 3 days. Transmission electron microscopy showed that about 5% of the mononuclear tumour cells exposed to NMF exhibited irregular bundles of thin (6–8 nm in diameter) and thick (12–15 nm in diameter) myofilaments (Figure 1e). These morphological features of rhabdomyogenic differentiation had never been observed in the mononuclear counterparts under standard growth conditions (Figure 1d) or after exposure to DMSO, HMBA and NaBut. The ultrastructural characteristics of the myotube-like giant cells observed after exposure to NMF, DMSO, HMBA and NaBut did not differ from those of their multinuclear

**Figure 1** Morphology of BA-HAN-1C tumour cells before and after differentiation induction: (**a**) small spindle-shaped mononuclear cells intermingled with few myotube-like giant cells under standard growth conditions as opposed to the larger, more flattened cells after exposure to NaBut (**b**) and the markedly elongated mononuclear cells after exposure to NMF (**c**). Electron microscopic detail of a mononuclear tumour cell in standard growth medium (**d**) lacking morphological features of rhabdomyogenic differentiation. Mononuclear tumour cell after exposure to NMF (**e**) exhibiting numerous irregular bundles of thick and thin myofilaments (star and inset). Arrows: myotube-like giant cells. **a**, **b**, **c**, bar = 100 μm; **d**, **e**, bar = 2 μm; inset, bar = 0.5 μm.

counterparts under standard growth conditions (Gerharz *et al.*, 1988). Non-specific cytotoxic effects were excluded by the trypan blue exclusion test and by transmission electron microscopy.

*Creatine kinase activity* There was a statistically significant increase ($P < 0.05$) in creatine kinase activity after exposure to polar compounds for 48 h when compared to the control ($21 \pm 4$ mU per $10^6$ cells). This increase in biochemical differentiation, however, proved to be reversible and the creatine kinase activity markedly decreased during the following days. After 120 h, only tumour cells exposed to NaBut (Figure 5) still exhibited a significantly ($P < 0.05$) elevated level of creatine kinase activity whereas the creatine kinase activity of cells exposed to NMF (Figure 3) and HMBA (Figure 4) did not differ significantly from the control. Tumour cells exposed to DMSO (Figure 2) for 120 h even showed a statistically significant ($P < 0.05$) decrease in creatine kinase activity.

*Fusion assay* The absolute number of myotube-like giant cells in cultures exposed to polar compounds for up to 96 h did not significantly differ from the control. Exposure to NMF for 120 h resulted in a marked increase in the absolute number of myotube-like giant cells. Because the fusion rate is cell density-dependent, the possibility had to be excluded that the increased number of myotube-like giant cells was only caused by a higher cell density in those cultures exposed to NMF. The relative frequency therefore of myotube-like giant cells, i.e. the ratio between the number of myotube-like giant cells (in 1/32 of the growth area of the culture flasks) and the total number of tumour cells per culture flask was calculated. For this ratio, a statistically significant increase ($P < 0.001$) was evident after 120 h.

Figure 2 Growth curves and creatine kinase activity of BA-HAN-1C tumour cells after exposure to DMSO. Each value represents the mean ± standard deviation of five replicate experiments. The creatine kinase activity is shown as a percentage of the control. 48 h and 120 h after exposure to DMSO, the creatine kinase activity is significantly different from the control ($P<0.05$; Wilcoxon test for unpaired samples). The difference between the growth curves is statistically significant ($P<0.001$; analysis of variance with two independent factors). ck, creatine kinase activity; $t_D$, mean doubling time; $n_{control}$, cell number in standard growth medium; $n_{DMSO}$, cell number in growth medium supplemented with DMSO.

Figure 3 Growth curves and creatine kinase activity of BA-HAN-1C tumour cells after exposure to NMF. Each value represents the mean ± standard deviation of five replicate experiments. The creatine kinase activity is shown as a percentage of the control. 48 h and 72 h after exposure to NMF, the creatine kinase activity is significantly different from the control ($P<0.05$; Wilcoxon test for unpaired samples). The difference between the growth curves is statistically significant ($P<0.001$; analysis of variance with two independent factors). ck, creatine kinase activity; $t_D$, mean doubling time; $n_{control}$, cell number in standard growth medium; $n_{NMF}$, cell number in growth medium supplemented with NMF.

In contrast, the absolute number of myotube-like giant cells in control cultures markedly exceeded the number of myotube-like giant cells in cultures exposed to DMSO, HMBA and NaBut after an incubation period for 120 h. The calculation of the relative frequency of myotube-like giant cells showed a statistically significant ($P<0.001$) decrease. (See Table II.)

*Assessment of proliferation*

*Growth rate* Under the conditions of our experiments, exposure to polar compounds always resulted in a statistically significant ($P<0.001$) inhibition of proliferation being most pronounced after 48 h. Detrimental effects of the polar compounds on cell viability were excluded by the trypan blue exclusion test. After 48 h, the proliferation in cultures exposed to polar compounds markedly accelerated. The initial growth inhibition observed after exposure to NMF was compensated for during the following days and after 120 h in culture, the cell density did not significantly differ between the control cultures and NMF-exposed cultures (Figure 3). In contrast, there was still a significantly lower cell density after 120 h($P<0.001$) in cultures exposed to DMSO (Figure 2), HMBA (Figure 4) and NaBut (Figure 5).

*Plating efficiency* Exposure to DMSO, HMBA, NaBut and NMF reduced the plating efficiency of BA-HAN-1C tumour cells rather heterogeneously. HMBA proved to be most effective, whereas NMF was least effective. (See Table III.)

### Discussion

The present study clearly demonstrates that DMSO, HMBA, NaBut and NMF are effective inducers of differentiation in our rhabdomyosarcoma cell line BA-HAN-1C, simultaneously inhibiting tumour cell proliferation. Nevertheless, the response of BA-HAN-1C tumour cells was markedly heterogeneous between the different polar compounds, probably indicating different mechanisms of action and signal transduction. Thus, the effects were most pronounced after exposure to NMF as evidenced by an increase in both biochemical and morphological differentiation and a significant increase in the proportion of terminally differentiated postmitotic myotube-like giant cells. In contrast, the effects of DMSO, HMBA and NaBut were exclusively confined to an increase in creatine kinase activity used as a biochemical marker of differentiation, whereas no increase in morphological differentiation was observed and the formation of terminally differentiated postmitotic mytotube-like giant cells even significantly decreased. A corresponding inhibition of myotube formation had already been reported for non-tumorigenic myoblast cell lines after exposure to DMSO, HMBA and NaBut (Miranda *et al.*, 1978; Blau & Epstein, 1979; Fiszman *et al.*, 1980; Endo & Nadal-Ginard, 1987). DMSO, HMBA and NaBut exhibited rather contradictory properties in our rhabdomyosarcoma cell line, inducing biochemical differentiation on the one hand and simultaneously inhibiting cell fusion and terminal differentiation on the other hand. These observations suggest

Figure 4 Growth curves and creatine kinase activity of BA-HAN-1C tumour cells after exposure to HMBA. Each value represents the mean ± standard deviation of five replicate experiments. The creatine kinase activity is shown as a percentage of the control. 48 h, 72 h and 96 h after exposure to HMBA, the creatine kinase activity is significantly different from the control ($P<0.05$; Wilcoxon test for unpaired samples). The difference between the growth curves is statistically significant ($P<0.001$; analysis of variance with two independent factors). ck, creatine kinase activity; $t_D$, mean doubling time; $n_{control}$, cell number in standard growth medium; $n_{HMBA}$, cell number in growth medium supplemented with HMBA.

Figure 5 Growth curves and creatine kinase activity of BA-HAN-1C tumour cells after exposure to NaBut. Each value represents the mean ± standard deviation of five replicate experiments. The creatine activity is shown as a percentage of the control. Between 24 and 120 h after exposure to NaBut, the creatine kinase activity is significantly different from the control ($P<0.05$; Wilcoxon test for unpaired samples). The difference between the growth curves is statistically significant ($P<0.001$; analysis of variance with two independent factors). ck, creatine kinase activity; $t_D$, mean doubling time; $n_{control}$, cell number in standard growth medium; $n_{NaBut}$, cell number in growth medium supplemented with NaBut.

**Table II** Fusion assay of BA-HAN-1C tumour cells after exposure to polar compounds

| | *Number of myotube-like giant cells** | | *Ratio* $\frac{\text{Number of myotube-like giant cells*}}{\text{total number of cells}}$ | |
|---|---|---|---|---|
| | *Initially* | *After 120 h* | *Initially* | *After 120 h* |
| Control | 2.4 ± 1.5 | 277 ± 64 | $8\times10^{-6}\pm5\times10^{-6}$ | $15\times10^{-6}\pm4\times10^{-6}$ |
| DMSO | 3.4 ± 1.5 | 23 ± 20 | $11\times10^{-6}\pm5\times10^{-6}$ | $7\times10^{-6}\pm5\times10^{-6}$ |
| HMBA | 3.6 ± 2.5 | 6 ± 5 | $12\times10^{-6}\pm8\times10^{-6}$ | $8\times10^{-7}\pm6\times10^{-7}$ |
| NaBut | 6.0 ± 3.5 | 5 ± 4 | $20\times10^{-6}\pm11\times10^{-6}$ | $8\times10^{-7}\pm5\times10^{-7}$ |
| NMF | 1.8 ± 0.8 | 959 ± 363 | $6\times10^{-6}\pm3\times10^{-6}$ | $83\times10^{-6}\pm33\times10^{-6}$ |

Each value represents the mean ± standard deviation of five replicate experiments. After 120 h the ratio between the number of myotube-like giant cells and the total number of cells is significantly different from the initial ratio for all experimental groups ($P<0.001$; analysis of variance with two independent factors; repeated measurements in one factor, i.e. time). * In 1/32 the growth area of the culture flask.

that the induction of biochemical differentiation by these differentiation inducers might be regulated independently from events leading to cell fusion and terminal differentiation. The mechanisms, however, by which polar compounds modulate cellular differentiation have not as yet been conclusively defined. NaBut has been shown to alter gene transcription by hyperacetylation of histones (Candido

**Table III** Plating efficiency of BA-HAN-1C tumour cells as a percentage of the control after exposure to polar compounds

| *Number of cells seeded per microwell* | *Plating efficiency (% of the control)* | | | |
|---|---|---|---|---|
| | *DMSO* | *HMBA* | *NaBut* | *NMF* |
| 10 | 98% | 4% | 91% | 99% |
| 1 | 53% | 2% | 27% | 82% |

*et al*., 1978) or by interfering with DNA methylation (Christman *et al*., 1980). Nevertheless, it is not yet known whether all the polar compounds exclusively act at the nuclear site altering gene transcription or epigenetically affect other cellular functions and structures such as glutathione metabolism (Bill *et al*., 1988) or cell membranes (Blau & Epstein, 1979; Meilhoc *et al*., 1986). Therefore, differentiation inducers such as DMSO, HMBA and NaBut that induce biochemical differentiation, but block the formation of terminally differentiated myotube-like giant cells, could become useful tools in further elucidating the mechanisms involved in differentiation induction by polar compounds.

It is important to note that induction of biochemical differentiation during the first 48 h was always accompanied by a statistically significant inhibition of tumour cell proliferation. However, the acceleration of proliferation beginning 48 h after exposure to polar compounds (Figures 2–5) was accompanied by a marked decrease of biochemical differentiation as indicated by the decline of the creatine kinase activity. The way in which proliferation and differentiation interact with each other, however, is still poorly understood. Interestingly in this context, cells of our rhabdomyosarcoma cell line BA-HAN-1C could also be induced to differentiate only by exposure to FCS-depleted medium that did not support cell proliferation (Gerharz *et al*., 1989). These observations might suggest a genetic programme interacting between proliferation and differentiation of tumour cells, which is similar to that proposed for normal cells (Harris *et al*., 1986; Sachs, 1987). This view is further supported by recent reports on the involvement of protooncogene expression in differentiation induction (Craig *et al*., 1984; Amatruda *et al*., 1985; Thiele *et al*., 1985; Mulder & Brattain, 1988). In our own investigations, we could demonstrate that the protooncogenes *fos* and *raf* are implicated in the process of differentiation induction in our rhabdomyosarcoma cell line BA-HAN-1C after exposure to retinoic acid (Doehmer *et al*., 1989). Further studies on biochemical and genetic levels are therefore in progress that should bring more insights into the regulatory events taking place in BA-HAN-1C tumour cells during differentiation induction by different types of differentiation inducers.

We would like to express our appreciation to Mrs C. Bürkner, Mrs A. Niederauer, Mrs K. Molter, Mrs H. Breitbach as well as to Mr K. Weber and Mr W. Meyer for their excellent technical assistance. We are grateful to Dr K.H. Schicketanz for his statistical evaluations. This work was supported by the Gesellschaft der Gönner und Förderer der Grundlagenforschung des Krebses.

### References

AMATRUDA, T.T., III, SIDELL, N., RANYARD, J. & KOEFFLER, H.P. (1985). Retinoic acid treatment of human neuroblastoma cells is associated with decreased n-*myc* expression. *Biochem. Biophys. Res. Commun.*, **126**, 1189.

AUGERON, C. & LABOISSE, C.L. (1984). Emergence of permanently differentiated cell clones in a human colonic cancer cell line in culture after treatment with sodium butyrate. *Cancer Res.*, **44**, 3961.

BILL, C.A., GESCHER, A. & HICKMAN, J.A. (1988). Effects of N-methylformamide on the growth, cell cycle, and glutathione status of murine TLX5 lymphoma cells. *Cancer Res.*, **48**, 3389.

BLAU, H.M. & EPSTEIN, C.J. (1979). Manipulation of myogenesis *in vitro*: reversible inhibition by DMSO. *Cell*, **17**, 95.

CANDIDO, E.P.M., REEVES, R. & DAVIE, J.R. (1978). Sodium butyrate inhibits histone deacetylation in cultured cells. *Cell*, **14**, 105.

CHRISTMAN, J.K., WEICH, M., SCHOENBRUN, B., SCHNEIDERMAN, N. & ACS, G. (1980). Hypomethylation of DNA during differentiation of Friend erythroleukemic cells. *J. Cell Biol.*, **86**, 366.

COLLINS, S.J., RUSCETTI, F.W., GALLAGHER, R.E. & GALLO, R.C. (1978). Terminal differentiation of human promyelocytic leukemia cells induced by dimethyl sulfoxide and other polar compounds. *Proc. Natl Acad Sci. USA*, **75**, 2458.

CORDEIRO, R.F. & SAVARESE, T.M. (1986). Role of glutathione depletion in the mechanism of action of *N*-methylformamide and *N,N*-dimethylformamide in a cultured human colon carcinoma cell line. *Cancer Res.*, **46**, 1297.

CRAIG, R.W., MAUE, R.J., HROMCHAK, R.A. & BLOCH, A. (1874). Decline of c-*myb* expression in human myeloblastic leukemia (ML-1) cells induced to differentiate with daunorubicin, conditioned medium, or retinoic acid. *Proc. Am. Assoc. Cancer Res.*, **25**, 64.

DELAPORTE, C., DAUTREAUX, B. & FARDEAU, M. (1986). Human myotube differentiation *in vitro* in different culture conditions. *Biol. Cell*, **57**, 17.

DEXTER, D.L. (1977). *N,N*-dimethylformamide-induced morphological differentiation and reduction of tumorigenicity in cultured mouse rhabdomyosarcoma cells. *Cancer Res.*, **37**, 3136.

DEXTER, D.L., CRABTREE, G.W., STOECKLER, J.D. & 5 others (1981). *N,N*-dimethylformamide and sodium butyrate modulation of the activities of purine-metabolizing enzymes in cultured human colon carcinoma cells. *Cancer Res.*, **41**, 808.

DEXTER, D.L., SPREMULLI, E.N., MATTOK, G.M., DIAMOND, J. & CALABRESI, P. (1982). Inhibition of the growth of human colon cancer xenografts by polar solvents. *Cancer Res.*, **42**, 5018.

DIBNER, M.D., IRELAND, K.A., KOERNER, L.A. & DEXTER, D.L. (1985). Polar solvent-induced changes in membrane lipid lateral diffusion in human colon cancer cells. *Cancer Res.*, **45**, 4998.

DOEHMER, J., GERHARZ, C.D., HOFFMAN, J.C., EDIGKAUFER, M., OESCH, F. & GABBERT, H. (1989). Altered expression of protooncogenes *fos* and *raf* accompanying differentiation in a rat rhabdomyosarcoma cell line (BA-HAN-1C) after exposure to retinoic acid. *Oncogenes* (in the press).

ENDO, T. & NADAL-GINARD, B. (1987). Three types of muscle-specific gene expression in fusion-blocked rat skeletal muscle cells: translational control in EGTA-treated cells. *Cell*, **49**, 515.

FISZMAN, M.Y., MONTARRAS, D., WRIGHT, W. & GROS, F. (1980). Expression of myogenic differentiation and myotube formation by chick embryo myoblasts in the presence of sodium butyrate. *Exp. Cell Res.*, **126**, 31.

FRESHNEY, R.I. (1985). Induction of differentiation in neoplastic cells. *Anticancer Res.*, **5**, 111.

FRIEND, C., SCHER, J., HOLLAND, G. & SATO, T. (1971). Hemoglobin synthesis in murine virus-induced leukemic cells *in vitro*: stimulation of erythroid differentiation by dimythelysulfoxide. *Proc. Natl Acad. Sci. USA*, **68**, 378.

GABBERT, H.E., GERHARZ, C.D., BIESALSKI, H.K., ENGERS, R. & LULEY, C. (1988*a*). Terminal differentiation and growth inhibition of a rat rhabdomyosarcoma cell line (BA-HAN-1C) *in vitro* after exposure to retinoic acid. *Cancer Res.*, **48**, 5264.

GABBERT, H.E., GERHARZ, C.D., ENGERS, R., MÜLLER-KLIESER, W. & MOLL, R. (1988*b*). Terminally differentiated postmitotic tumor cells in a rat rhabdomyosarcoma cell line. *Virchows Arch. B (Cell Pathol.)*, **55**, 255.

GARVIN, A.J., STANLEY, W.S., BENNETT, D.D., SULLIVAN, J.J. & SENS, D.A. (1986). The *in vitro* growth, heterotransplantation, and differentiation of a human rhabdomyosarcoma cell line. *Am. J. Pathol.*, **125**, 208.

GERHARZ, C.D., GABBERT, H., MOLL, R., MELLIN, W., ENGERS, R. & GABBIANI, G. (1988). The intraclonal and interclonal phenotypic heterogeneity in a rhabdomyosarcoma cell line with abortive imitation of embryonic myogenesis. *Virchows Arch. B (Cell Pathol.)*, **55**, 193.

GERHARZ, C.D., GABBERT, H.E., BIESALSKI, H.K., ENGERS, R. & LULEY, C. (1989). Fetal calf serum and retinoic acid affect proliferation and terminal differentiation of a rat rhabdomyosarcoma cell line. *Br. J. Cancer*, **59**, 61.

HARRIS, C.C., YOAKUM, G.H., LECHNER, J.F. & 5 others (1986). Growth differentiation, and neoplastic transformation of human bronchial epithelial cells. In *Biochemical and Molecular Epidemiology of Cancer*, p. 213. Alan R. Liss: New York.

IWAKAWA, M., TOFILON, P.J., HUNTER, N., STEPHENS, L.C. & MILAS, L. (1987). Antitumor and antimetastatic activity of the differentiating agent *N*-methylformamide in murine tumor systems. *Clin. Exp. Metastasis*, **5**, 289.

LEE, K.-L., PETKOVICH, P.M. & HEERSCHE, J.N.M. (1988). The effects of sodium butyrate on the retinoic acid-induced changes in 1,25-dihydroxyvitamin $D_3$ receptors in tumorigenic and non-tumorigenic bone derived cell lines. *Endocrinology*, **122**, 2399.

MANSFIELD, B.K., MANN, R.C. & SELKIRK, J.K. (1988). Two-dimensional gel electrophoretic analysis of cytoplasmic proteins from Friend erythroleukemia cells chemically induced to undergo terminal erythroid differentiation. *Cancer Res.*, **48**, 1110.

MEILHOC, E., MOUTIN, M.-J. & OSBORNE, H.B. (1986). Catabolites produced by the deacetylation of hexamethylenebisacetamide play a key role in murine erythroleukaemic-cell differentiation. *Biochem. J.*, **238**, 701.

METCALF, D. (1983). How many cancers are reversible or suppressible? *Pathology*, **15**, 1.

MIRANDA, A.F., NETTE, E.G., KHAN, S., BROCKBANK, K. & SCHONBERG, A. (1978). Alteration of myeloblast phenotype by dimethyl sulfoxide. *Proc. Natl Acad. Sci. USA*, **75**, 3826.

MULDER, K.M. & BRATTAIN, M.G. (1988). Alteration in c-*myc* expression in relation to maturational status of human colon carcinoma cells. *Int. J. Cancer*, **42**, 64.

NORDENBERG, J., WASSERMAN, L., PELED, A., MALIK, Z., STENZEL, K.H. & NOVOGRODSKY, A. (1987). Biochemical and ultrastructural alterations accompany the anti-proliferative effect of butyrate on melanoma cells. *Br. J. Cancer*, **55**, 493–497.

PIERCE, G.B. (1974). The benign cells of malignant tumors. In *Development Aspects of Cancerogenesis and Immunity*, King, P.J. (ed.) p. 3. Academic Press: New York.

PRASAD, K.N. & SINHA, P.K. (1976). Effect of sodium butyrate on mammalian cells in culture: a review. *In Vitro*, **12**, 125.

RAMSAY, R.G., IKEDA, K., RIFKIND, R.A. & MARKS, P.A. (1986). Changes in gene expression associated with induced differentiation of erythroleukemia: protooncogenes, globin genes, and cell division. *Proc. Natl Acad. Sci. USA*, **83**, 6849.

REUBEN, R.C., WIFE, R.L., BRESLOW, R., RIFKIND, R.A. & MARKS, P.A. (1976). A new group of potent inducers of differentiation in murine erythroleukemia cells. *Proc. Natl Acad. Sci. USA*, **73**, 862.

SACHS, L. (1987). Cell differentiation and bypassing of genetic defects in the suppression of malignancy. *Cancer Res.*, **47**, 1981.

SARTORELLI, A.C. (1985). Malignant cell differentiation as a potential therapeutic approach. *Br. J. Cancer*, **52**, 293.

SNYDER, S.W., EGORIN, M.J., GEELHAAR, L.A., HAMBURGER, A.W. & CALLERY, P.S. (1988). Induction of differentiation of human pro-myelocytic leukemia cells (HL60) by metabolites of hexamethylene bisacetamide. *Cancer Res.*, **48**, 3613.

SPREMULLI, E.N. & DEXTER, D.L. (1984). Polar solvents: a novel class of antineoplastic agents. *J. Clin. Oncol.*, **2**, 227.

TARELLA, C., FERRERO, D., GALLO, E., PAGLIARDI, G.L. & RUSCETTI, F.W. (1982). Induction of differentiation of HL-60 cells by dimethyl sulfoxide: evidence for a stochastic model not linked to the cell division cycle. *Cancer Res.*, **42**, 445.

THIELE, C.J., REYNOLDS, C.P. & ISRAEL, M.A. (1985). Decreased expression on n-myc orecedes retinoic acid-induced morphological differentiation of human neuroblastoma. *Nature*, **313**, 404.

TSAO, D., MORITA, A., BELLA, JR, A., LUU, P. & KIM, Y.S. (1982). Differential effects of sodium butyrate, dimethyl sulfoxide, and retinoic acid on membrane-associated antigen, enzymes, and glycoproteins of human rectal adenocarcinoma cells. *Cancer Res.*, **42**, 1052.